# The Ecological–Evolutionary Game of the Insect Gut Microbiome: Environmental Drivers, Host Regulation, and Prospects for Cross-Cutting Applications

**DOI:** 10.3390/vetsci12090866

**Published:** 2025-09-05

**Authors:** Ying Wang, Jie Tang, Yao Chen, Shuyi Chen, Sumin Chen, Xin Yu, Caijing Wan, Guoqi Xiang, Yaping Chen, Qiang Li

**Affiliations:** 1 Key Laboratory of Coarse Cereal Processing, Ministry of Agriculture and Rural Affairs, Sichuan Engineering & Technology Research Center of Coarse Cereal Industrialization, School of Food and Biological Engineering, Chengdu University, Chengdu 610106, China; 2 Yunnan Plateau Characteristic Agricultural Industry Research Institute, Yunnan Agricultural University, Kunming 650500, China; 3 Key Laboratory of Basic Research and Application Promotion of Solar Energy Technology in Higher Education Institutions of Sichuan Province, Panzhihua University, Panzhihua 617000, China

**Keywords:** insect gut microbes, microecosystems, host–microbe interactions, immune regulation, biotechnology applications

## Abstract

The insect gut contains a complex and diverse microbial community, and the composition of the insect gut microbial community is influenced by multiple factors such as the host’s genetics, dietary habits, and the external environment. The host’s immune system maintains the stability and balance of the microbial community through a number of mechanisms. The microorganisms in this community play key roles in the nutrient metabolism, detoxification, immune regulation, development, and behaveior of insects. In recent years, the relevant literature has reported advances in the study of insect gut microbes, indicating the potential applications of insect gut microbes in several fields. The aim of this review is to provide a comprehensive overview of the current information on the structure of insect gut microbial communities and complex host–microbe–environment interactions. The diversity of insects’ gut microbial communities and the functions of their gut microbes are revealed. By studying insect gut microbial communities, we can gain insights into the functions of these microbes in the host and explore the causal relationships between them and the host’s physiology and behavior. This will not only help us to understand the mechanism of action of the microbiome, but also provide a basis for the development of innovative biotechnology based on insect gut microbes. This research has significant theoretical value in academia and also has a wide range of applications in agriculture, environmental protection, industrial production, and healthcare.

## Simple Summary

The diversity, function, and complex interactions of insect gut microbiota with their hosts and environments. The composition of insect gut microbiota is influenced by a combination of host genetics, diet, and environmental factors, and is finely regulated by the host immune system. These microorganisms play a critical role in nutrient metabolism, detoxification, immune regulation, and host development and behavior. Recent studies have shown that insect gut microbiota not only hold significant importance in basic theoretical research but also demonstrate broad application potential in fields such as agriculture, environmental protection, industrial biotechnology, and healthcare. In-depth exploration of this field will provide a crucial foundation for understanding host-microbiota interaction mechanisms and developing novel biotechnologies.

## 1. Introduction

Recently, there has been increasing attention to the role of the insect gut microbiome in host health, immunity, and metabolism. The gut microbiota is a microbial population inhabiting the gastrointestinal tract and has profound effects on the physiological processes of the host, including digestion and immunity. The study of insects, as a diverse and ecologically significant group of species, provides unique perspectives on host–microbiome interactions and their evolutionary impacts. Studies of the insect gut microbiota have provided valuable insights into microbial stability, immune regulation, and mechanisms of adaptation to environmental changes.

The fruit fly Drosophila melanogaster serves as an important model organism for gut microbiome research, in which intestinal microbes critically regulate both host immunity and metabolism. Buchon et al. pointed out the complex interactions between the gut microbiota and the host immune system, and studies suggest that the microbiota is capable of modulating the intestinal morphology and gene expression and plays an important role under environmental stress [1]. Their study also revealed the role of immune signaling pathways in changes in host physiology, highlighting the relationship between the insect host and its gut microbiota. K.A. Lee and W.J. Lee further explored immune–metabolic interactions in *Drosophila* during systemic and intestinal infections, revealing how the immune system’s response to microbial pathogens affects metabolic processes such as energy production and storage [2]. Research shows that the insect gut regulates the composition of the microbial community through selective filtration mechanisms, maintaining beneficial bacteria and eliminating pathogens. This process plays a key role in maintaining the gut microecological balance and host health [3]. It has also been shown that this filtering mechanism acts as a structural barrier to organize the microbiota into different functional regions, thereby improving the host’s ability to cope with microbial perturbations. Bai et al. further explored the role of regulatory proteins in maintaining microbial homeostasis and investigated their effects on insect health and development [4]. Research shows that DUOX prevents chronic inflammation by maintaining the intestinal integrity and promoting immune tolerance [5]. The gut microbiota not only participates in nutrient metabolism, but also plays an important role in barrier maintenance, resistance to environmental stress, and pathogen defense. Research shows that environmental factors (such as diet, heavy metals, and microplastics) significantly affect the composition of insect gut microbiota. Microbial signals such as quorum sensing molecules participate in immune regulation [6]. Exposure to pollutants may disrupt the balance of microbial communities, thereby having negative effects on host health and behavior, highlighting the importance of environment–microbe–host interactions.

In summary, the insect gut microbiota is a complex and dynamic system with a crucial role in host health, immunity, and ecological interactions. As research progresses, there is growing evidence that the microbiota is not merely an appendage of the host, but actively participates in shaping the host physiology and behavior. The gut microbiota plays a variety of roles in processes such as immunomodulation, detoxification, microbiota regulation, and adaptation to environmental stresses. Further research will offer new insights and strategies for improving insect health, managing pests, and utilizing microbiota in biotechnology applications.

## 2. The Structural Features and Microbial Diversity of the Insect Gut

### 2.1. The Anatomical Structure and Microenvironment of the Insect Gut

**Figure 1:**
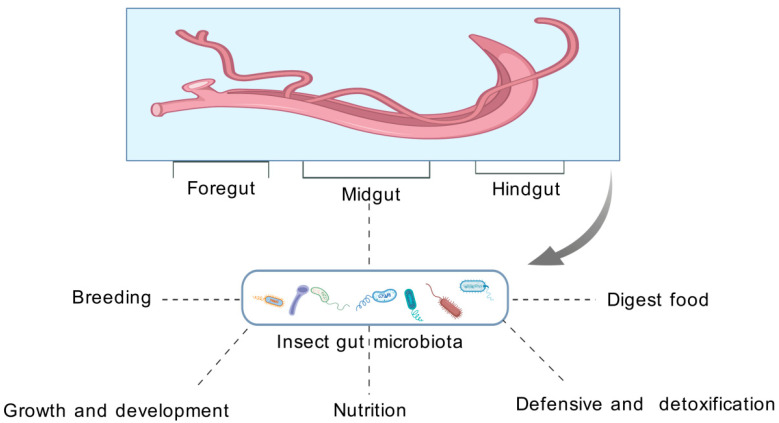
Dynamic regulation and functional evolution of the insect gut microbiota during host development (illustrates the dynamic roles of insect gut microbes at different stages of development). The insect gut is divided into the foregut, midgut, and hindgut, and microbes in each region perform specific functions at different stages. This graphic was generated using the General-Purpose High-Quality Biomedical Graphics Database (GDP), a comprehensive repository of standardized biomedical visualization tools [17].

The insect gut is a complex and functionally diverse organ system, whose unique anatomy and microenvironment provide an ideal habitat for abundant microbial communities. Lin et al. showed that, from the perspective of its developmental origin and functional features, the insect gut is divided into three major regions: the foregut, midgut, and hindgut. This structural division reflects its functional divergence during evolution [7]. Broderick et al. conducted a thorough study of Drosophila melanogaster and found that the foregut and hindgut originate from the ectoderm. The inner wall of these structures has a chitin layer, which is mainly responsible for food storage and water reabsorption. On the other hand, the midgut originates from the endoderm and is the main site of digestion and absorption. These histological characteristics are highly related to their functions [8]. As shown in Figure 1

As the core region of the insect digestive system, the midgut has a unique organizational structure. A review by Engel and Moran systematically elaborated on the structural characteristics of the midgut, indicating that its epithelium consists of a single layer of columnar cells. The surface is covered with a special structure called the peritrophic membrane, which has important physiological significance in the insect digestive system [9]. Through morphological and molecular characterization studies, Buchon et al. found that the peritrophic membrane is a translucent network composed of chitin fibers, glycoproteins, and proteins that play multiple roles in food digestion and protection of the intestinal epithelium [10]. Their study specifically highlighted the critical role of the peritrophic membrane in maintaining intestinal homeostasis, not only preventing mechanical damage but also selectively regulating substance exchange.

The characteristics of the intestinal microenvironment are regulated by multiple factors, and a study by Neyen et al. revealed complex interactions of key factors, including the pH, oxygen concentration, osmolality, and nutrient distribution [11]. In a systematic study of Lepidoptera, Paredes et al. found that the pH of different intestinal segments varies significantly, with the anterior portion of the midgut usually being strongly alkaline (pH 10–11) and the posterior portion tending to be neutral. This pH gradient not only affects the activity of digestive enzymes, but also provides a diverse habitat for microorganisms with different pH adaptations, forming a unique microcosm [12]. In terms of the dynamic regulation of the microenvironment, Ryu et al. showed a gradual gradient of the oxygen concentration in the gut from the intestinal wall to the lumen, forming a continuous environment that varied from aerobic to slightly aerobic to anaerobic. This gradient distribution is important for maintaining the diversity of microbial communities and can provide a suitable living space for microorganisms with different oxygen requirements [13]. In particular, Ha et al. found in their Drosophila research that the gut has a unique immune microenvironment. Intestinal epithelial cells can actively secrete various antimicrobial peptides and reactive oxygen species (ROS), as well as other defensive substances. This immune regulatory mechanism plays a central role in maintaining the balance of intestinal microbes [14].

Recent studies have also revealed the distribution characteristics of nutrients in the gut. Cole et al. found that simple sugars were mainly absorbed in the foregut and anterior midgut, while amino acids and fatty acids were absorbed in the posterior gut and posterior midgut. This heterogeneous spatial distribution provided a specific niche for microbial communities with different functions [15]. The role of this material distribution pattern in shaping the microbial community structure was highlighted as an important basis for ensuring the stability of insect gut microecosystems [16].

This highly organized structure provides the basis for maintaining a stable commensal microbial community, while also providing effective defense mechanisms against pathogen invasion. Recent studies have shown that the structural features and microenvironmental regulatory mechanisms of the insect gut are the result of long-term evolution, reflecting the coevolutionary relationship between the host and the microbe. Future studies should further focus on the dynamic changes in microenvironmental factors and their mechanisms regulating the microbial community’s structure and function, especially in the context of global environmental change. An in-depth understanding of these mechanisms is important for predicting and regulating insect–microbe interaction relationships. Figure 1. Structure and microenvironment of the insect gut: evolutionary adaptations for microbial colonization and functional maintenance.

### 2.2. The Composition of the Insect Gut Microbial Community

Insect gut microbial communities exhibit unique diversity and complexity. In the study of black soldier fly, *Hermetia illucens*, larvae, Daniele et al. found that the composition of the intestinal microbial community was significantly affected by the diet and the intestinal region. Different sections of the intestine showed specific microbial distribution characteristics [18]. This review study further revealed that these microorganisms mainly included bacteria, fungi, archaea, and viruses, with bacteria being the most dominant and widely studied group [19]. As shown in Table 1. The evolution of insects has led to highly specialized host structures, including specialized bacterial cls, symbiotic organs, and microhabitats, which provide obligate symbionts with a stable living environment and the necessary nutritional support. The main obligate symbiotic microbial communities include the phyla Proteobacteria, Bacteroidetes, Firmicutes, Actinobacteria, Spirochaetes, and Verrucomicrobia [20].

**Table 1:** Common phyla in gut microbiota isolated from different insects, associated microorganisms found in different insect orders, and their functions.

Insect Classification	Representative Species		Microbial Genus	Relative Abundance (%)
Isoptera	Black-winged termite		Treponema Bacteroides	35–60% 20–45%
Order Lepidoptera	Fall armyworm		Enterococcus	25–50%
Order Hymenoptera	Bees		Gilliamella	15–30%
Order Coleoptera	Red-bellied woodpecker		Lactobacillus	20–40%
Order Diptera	Black-bellied fruit fly		Acetobacter Lactobacillus	10–20% 5–15%
Order Orthoptera	Desert locust		Weissella	30–45%
Order Hemiptera	Aphid		Buchnera	90%
Order Odonata	Green dragonfly larva		Comamonas	20–35%
Order Blattodea	Blattodea		Blattabacterium	70–80%
Order Phthiraptera	Body lice		Riesia	95%
Primary Functions		Representative species		Citation
Wood cellulose degradation, nitrogen fixation Hemicellulose decomposition		Biomass energy conversion, soil improvement		[21]
Biodegradation of microplastics		Environmental management		[22]
Pollen polysaccharide metabolism		Improving agricultural pollination efficiency		[23]
Pesticide (pyrethroid) degradation		Green control of agricultural pests		[24]
Ethanol metabolism, lifespan regulation		Human intestinal disease model		[25]
Cellulose digestion, group pheromone synthesis		Development of locust control strate gies		[26]
Essential amino acid synthesis		Water pollution biological monitor ing		[27]
Aquatic heavy metal (Cd/Pb) chelation		Biological monitoring of water pollution		[28]
Degradation of organochlorine pesticides		Urban pest control		[29]
Vitamin B synthesis		Control of animal and human parasitic disease		[30]

Through the study of herbivorous insects, Shan et al. found that Proteobacteria (Pseudomonas) and thick-walled bacteria (*Bacillus*) are usually the most dominant groups in bacterial communities. This dominance may be closely related to the evolutionary history and ecological adaptation of the host [31]. In particular, Yang et al., in studying plastic-degrading insects, found that certain bacterial taxa with special functions, such as *Pseudomonas* and *Bacillus*, play important roles in degrading complex organic substances [32].

In terms of the dynamic changes in microbial communities, it has been shown that environmental factors such as the temperature, humidity, and pollutants can significantly affect the composition and structure of microbial communities [33]. Insect gut microbiota represent a potential resource for biofuel production, with their unique enzyme systems capable of efficiently degrading lignocellulose (e.g., termite and beetle gut microbiota). Research is focusing on the identification and modification of key microorganisms and enzymes to enhance the biomass conversion efficiency. These findings not only reveal the ecological functions of microorganisms but also provide new insights for sustainable energy development [34]. Future research can further analyse the functional interaction mechanisms of microbial communities through multi-omics technologies and promote their application in synthetic biology [35].

These research results not only deepen our understanding of the composition of insect gut microbial communities, but also provide an important basis for understanding the ecological function and evolutionary mechanisms of microbial communities. Future studies are required to further explore the regulatory mechanisms of the microbial community composition and the interaction relationships among different microbial taxa.

### 2.3. Factors That Influence the Microbial Community Structure

The assembly of the insect gut microbiota is a complex process driven by multiple factors, including host genetics, developmental stage, and environmental exposure [36]. This finding provides an important basis for understanding the role of nutritional factors in shaping microbial communities. In the study of polystyrene biodegradation, the genetic background of the host is a fundamental factor that influences the microbial community structure. Different insect species, and even different genotypes of the same species, may lead to significant differences in the microbial community composition [37]. Further research suggests that host genetics may exert regulatory effects on the microbial community through pathways such as modulating the intestinal environment, immune response, and metabolic profile [38]. The importance of environmental factors was well demonstrated by Zhang et al. They found that the nanoplastic photoaging process affected the gut microbial community structure of aquatic insect chironomids, *Chironomus kiiensis*, mainly by disrupting their gut integrity and changing the composition of the microbial community [39]. Transitions between developmental stages are another key influencing factor. When studying the pink cotton bollworm, Sun et al. found that the insect’s development from the larval stage to an adult was accompanied by significant remodeling of the gut microbial community [40]. Research indicates that environmental factors, such as the temperature and humidity, could indirectly affect microbial communities by altering the physiological state of the host [41]. This remodeling may be related to developmental changes in the intestinal structure, physiological function, and the immune system. Yanchun et al. further showed that these developmentally relevant changes in the microbial community have important implications for the host’s physiological functions [42].

Microbial interactions also play an important role in the formation of the community structure. Studying the interactions between plants and microorganisms, Yuxin et al. found that certain microorganisms may affect the structure of other communities by producing metabolites or competing for nutrients [43]. This finding confirms that interactions between microorganisms are a key mechanism for maintaining the stability of community ecological function and structure. It is noteworthy that these influencing factors do not act independently. Existing research indicates that there are complex interactions between the host and the microbial community, and these interactions collectively drive the dynamic succession of the microecosystem [44]. Elucidating the relative contributions of these factors and their interaction mechanisms is of great value for predicting and targeting the regulation of the insect gut microbiota structure, providing a theoretical basis for the development of green pest control strategies based on microbiome management.

## 3. Functions of Insect Gut Microbes

### 3.1. Function of Nutrient Metabolism

The gut microbiota of an insect forms interacts closely with its host. By encoding a rich metabolic enzyme system, it directly participates in the host’s nutritional metabolism process, significantly enhancing the insect’s adaptability to different food sources [45]. This finding provides new insights allowing us to understand the mechanisms of nutritional adaptation in insects. In their study on wild pollinating insects, Jilian et al. revealed that the gut microbial community plays a decisive role in the degradation of complex carbohydrates. They found that certain microorganisms are able to secrete a variety of glycoside hydrolases, such as cellulase and xylanase. The synergistic action of these enzymes can convert indigestible plant polysaccharides into monosaccharides that the host can absorb [46]. Further analysis using metagenomics and metabolomics revealed that this degradation process relies on an interactive network composed of multiple microbial groups, which work together through functional complementarity to break down and convert complex polysaccharides [47]. In terms of nitrogen metabolism, Yaxin et al.’s study of black gadfly, *Hermetia illucens*, larvae found that certain intestinal bacteria have a nitrogen-fixing capacity and are able to convert atmospheric nitrogen into ammonia, providing an additional source of nitrogen for the host [48]. When the nitrogen sources in food are limited, the key role of nitrogen-fixing microorganisms becomes apparent. Research by McMillan’s team has confirmed that plants can actively regulate the composition of insect gut microbiota, thereby altering the host’s nitrogen metabolism pathways, demonstrating a cross-border nutritional regulation system [49]. Insects and their gut microbiota interact closely to effectively promote the biological conversion of agricultural food waste. The relevant microorganisms not only support the growth and development of insects, but also directly participate in the decomposition and conversion of organic waste [50]. Yuan Qi and colleagues found in a review of recent research that pathogenic fungi and gut microbiota interact to induce host death, and that gut microbiota with significant effects on the host can participate in pest control. Advances in genome editing technology are also new to the field of pest management [51]. The digestibility of cellulose and hemicellulose was determined for four species of grasshoppers, and the relationship between digestibility and gut microbial diversity was analysed. This study provides foundational data for the development of digestible bioreactors for cellulose and hemicellulose, and may offer new insights into straw degradation [52]. This highly efficient interaction mechanism between insects and their gut microbiota also provides both theoretical underpinnings and practical potential for developing agricultural waste recycling processes based on microbiome technology [53].

These findings not only deepen our understanding of microbial trophic interactions in insects, but also provide a theoretical basis for the development of microbial-based biotechnological applications. Understanding these complex metabolic networks is important for improving the nutritional status of beneficial insects or for developing novel bioenergy technologies.

### 3.2. Detoxification Effect

The detoxification of insect gut microbes is a remarkable area of research. In grasshoppers, *Eucriotettix oculatus*, Xiao-Dong et al. found that gut microbial communities can respond to heavy metal pollution and enhance the host tolerance by changing the community composition and diversity [54]. This adaptive alteration reflects the plasticity of microbial communities under environmental stress. Meiki et al. developed sterile insect culture methods while studying leaf beetles, and their study revealed the critical role of gut microbes in the degradation of plant secondary metabolites [55]. A review by Sandipan et al. further states that certain insect gut microbes are capable of producing specific enzymes that convert toxic compounds into less toxic or non-toxic forms [56]. This detoxification mechanism allows insects to adapt to plant foods containing natural toxins. When studying *Bacillus thuringiensis*, Siyi et al. found that certain intestinal bacteria not only participate in the degradation of environmental pollutants, but also enhance the host’s detoxification ability by producing specific metabolites [57]. Lau et al. showed that the detoxification of gut microbes is often closely related to the immune regulation function, and this multiple-action mechanism improves the host’s ability to cope with environmental stresses [58]. Through termite transcriptome analysis, YaLing et al. found that pathogenic fungal infection can significantly alter the structure of the intestinal microbiota, and this community restructuring may be an adaptive strategy for the host to enhance their disease resistance [59]. These findings not only deepen our understanding of insect–microbial interactions, but also provide new ideas for the development of microbe-based bioremediation techniques.

### 3.3. Immunomodulatory Effects

The regulatory role of insect gut microbes in the host immune system is complex and delicate. This immunomodulatory function reflects the result of long-term coevolution between microbes and their host. In their study of plastic-degrading superbugs, Lu et al. found that gut microbial communities are remarkably ecologically robust and able to maintain host immune homeostasis in the face of environmental stresses such as antibiotics and microplastics [60]. This adaptive mechanism is crucial for the survival of insects in complex environments. Further research has revealed that certain gut microbes can regulate the host’s metabolism by producing specific microbial proteases [61]. By examining trophic-level delivery of metal oxide nanoparticles, Wang et al. found that changes in the gut microbial community impact oxidative stress and immune responses in the host [62]. Morimura et al. observed in the grapevine pest *Sapolygus spinolae* that the seasonal dynamics of the gut microbial community are closely related to the immune defenses of the host [63]. The role of probiotics in enhancing the immune system of honeybees was particularly emphasized in a study by Robino et al., who found that the addition of specific probiotic strains significantly increased hemolymphocyte activity and phenoloxidase activity [64]. These findings deepen our understanding of insect–microbe immune interactions and provide new ideas for further development of microbe-based biocontrol strategies. This complex immunomodulatory network demonstrates the important role of microbial communities in maintaining host health.

### 3.4. Developmental and Behavioral Effects

Research indicates that the gut microbiota of insects regulates the development and behavioral patterns of its host through complex mechanisms. Taking honeybees (*Apis mellifera*) as an example, changes in the composition of the gut microbiota are closely related to differentiation in labor behavior, suggesting that microbial communities may regulate the behavioral characteristics of social insects through neuroendocrine or immune pathways [65]. This discovery provides a new research direction for explaining the evolutionary mechanisms of insect social behavior. In terms of host development, environmental stressors such as pesticide exposure can have long-term effects on the gut microbiota. Experimental evidence shows that exposure to sublethal doses of thiamethoxam during the larval stage in bees leads to persistent changes in the gut microbiota structure during the adult stage, and this microbiota disruption is significantly associated with abnormal host growth and development [66]. This phenomenon reveals the cascading interactions between environmental stress, the microbiota, and host development. It is worth noting that the microbiota exhibits significant specificity in regulating the host behavior. Studies have found that although the gut microbiota can significantly influence the frequency of feeding behavior, its impact on related characteristic metabolic markers is relatively limited [67]. Plant-growth-promoting rhizobacteria (PGPR) inhibit pest development by regulating the intestinal microbiota of insects, providing a new approach to green pest control. Further research is needed to elucidate the interaction mechanisms and optimize application technologies [68]. The regulation of development and behavior often requires interactions at multiple levels. These discoveries have advanced our understanding of insect–microbe interactions and set the theoretical basis for developing strategies for behavioral regulation based on that performed by microbes. Future research is essential to uncover the molecular mechanisms of microbe-mediated developmental and behavioral regulation, as well as to investigate the conservation and specificity of these mechanisms across different insect taxa.

## 4. Interaction of the Insect Gut Immune System and the Microbial Community

### 4.1. The Evolutionary Immune Function of Insect Pattern Recognition Receptors

Pattern recognition receptors (PRRs) play a central role in the insect immune system. Phylogenetic studies of Lepidoptera indicate that understanding the molecular mechanisms of their evolution holds significant importance for both fundamental biology and the silk industry. This co-evolutionary relationship reflects the process by which insects adapt to diverse pathogens [69]. Through the in-depth study of peptidoglycan recognition proteins (PGRPs), research has shown that these receptors, as regulatory switches of the innate immune system, can accurately identify different types of pathogenic microorganisms and activate the corresponding immune response using a specific binding mode [70]. In terms of model organism research, Pereira et al. systematically elaborated on the mechanism of the role of PRRs in pathogen recognition and immune response initiation by using the large wax moth, *Galleria mellonella*, as an infection model. In particular, they highlighted the differential response patterns that these receptors exhibit in identifying both Gram-positive and Gram-negative bacteria [71]. Further exploring this direction, Wang and his colleagues used insect models to study a new generation of antimicrobial agents, confirming the key role of pattern recognition receptors (PRRs) in pathogen recognition and immune responses, which is of great significance for the development of future antimicrobial treatments [72]. Hoffmann et al. elaborated on the evolutionary features of PRRs and found that these receptors are highly conserved among invertebrates. This conservation implies their important role in innate immunity [73]. Subsequently, Silverman and Maniatis found, through a comparative study, that there are significant similarities between the PRR signaling pathways in insects and mammals, particularly in the mechanism of activation of the NF-κB pathway. This finding provides important clues for understanding the evolution of the immune system [74]. In a functional study, Wand and his team analyzed in detail the complex interactions between *Klebsiella pneumoniae* and the host immune system with the help of a wax moth infection model. It was shown that pattern recognition receptors (PRRs) are critical for pathogen recognition and initiation of immune defense. These receptors not only specifically detect the presence of the pathogen, but also trigger a series of complex, hierarchical immune responses in response to infection [75]. In their study on Aedes aegypti, Wang et al. discovered a close connection between the activation of prephenoloxidase-3 and the recognition of PRRs. This discovery offers new perspectives on how an insect’s antifungal immunity functions [76].

These findings deepen our understanding of the functions of insect PRRs and provide a theoretical basis for the development of novel biological control strategies. Future studies are needed to further explore the molecular recognition mechanisms of PRRs, especially how to coordinate the activation of multiple immune response pathways and the interactive regulatory network between these pathways.

### 4.2. Insect Immunology: Signal Networks in Biological Control

Insect immune signaling pathways are complex and sophisticated regulatory networks. By studying pea aphids, Xu et al. found that the phenol oxidase system plays a key role in resistance to bacterial and fungal infections and that this immune response involves the synergistic activation of multiple signaling pathways [77]. It has been shown that phenoloxidases are not only involved in the recognition and clearance of pathogens, but also play an important role in the amplification of immune responses. The biological significance of blood cells in immune signaling was further explored by Stanley et al. They found that blood cells are not only the executors of immune effects but are also able to regulate the activity of the entire immune network through the secretion of various signaling molecules [78]. A study by Zhang et al. provides insight into the mechanism of action of antimicrobial peptides, revealing how these effector molecules are activated through different signaling pathways and perform multiple functions in immune defense [79]. During the activation of the Toll and IMD signaling pathways, the expression of antimicrobial peptides is intricately regulated to ensure the precision and effectiveness of the immune response. Hetru and Hoffmann delved into the role of NF-κB in the immune response of Drosophila through a detailed study. They found that this transcription factor plays a central role in integrating different immune signaling pathways [80].

A groundbreaking study by Leulier et al. demonstrated that the Drosophila immune system detects bacteria through specific peptidoglycan recognition. This finding provides crucial insights for comprehending how pattern recognition receptors activate downstream signaling pathways [81]. Their study places particular emphasis on the specificity of signal transduction, i.e., different pathogen recognition patterns activate different signaling pathways. Wang et al., in their study of the Taiwan termite, identified a key role for prephenol oxidase in the blackening process and bacterial defense, a finding that demonstrates interactive regulation between signaling pathways [82]. Studies on the Asian corn borer by Prabu et al. revealed the relationship between the phenol oxidase activation mechanism and resistance to *Bacillus thuringiensis* and elucidated how immune signaling pathways regulate insects’ resistance to biopesticides [83]. This study deepens our understanding of the function of immune signaling pathways and also provides new ideas for improved pest control strategies. In their review, Wang et al. highlighted the importance of antimicrobial peptides in the post-antibiotic era, pointing out the potential for developing novel antimicrobial strategies based on studies of immune signaling pathways [84].

These results demonstrate the complexity and precision of insect immune signaling pathways and also provide a theoretical basis for the development of novel biological control methods. Future studies are needed to further explore the synergistic mechanisms between different signaling pathways and the influence of environmental factors on immune signaling networks. This has important practical implications for improving agricultural production and disease prevention and control.

### 4.3. Production and Function of Antimicrobial Peptides (AMPs)

Antimicrobial peptides (AMPs) are important immune effector molecules in insects. Research conducted by Cerenius et al. revealed how the prophenoloxidase system functions in invertebrate immunity and its close connection to the generation of AMPs, forming a robust defense mechanism [85]. This discovery helps us better understand how different aspects of the insect immune system collaborate to defend against threats. By conducting a deep investigation into the mechanism of action of pattern recognition receptors, Takeuchi and Akira revealed the upstream regulatory network of AMP production, demonstrating the close relationship between the inflammatory response and the expression of antimicrobial peptides [86]. When studying fungal immune recognition, Patin et al. found that specific pattern recognition receptors can trigger the production of antifungal antimicrobial peptides. This specific response enhances the host defense against various pathogens [87]. A systematic study by Zhang et al. further elucidates the regulatory molecular and cellular pathways involved in the insect antimicrobial immune response, with particular emphasis on the centrality of antimicrobial peptides in innate immunity [88]. Research has found that there is a synergistic effect between capsular polysaccharide-specific antibody responses and antimicrobial peptides (AMPs), revealing the regulatory function of the antimicrobial peptide system in the complement cascade reaction [89]. Swaminathan et al. found that peptidoglycan recognition proteins regulate the production of antimicrobial peptides through dual regulation. This precise regulation ensures the specificity and effectiveness of the immune response [89]. Jiang et al.’s research on the tobacco moth demonstrated that βGRP-2, an acute-phase protein, recognizes both β-1,3-glucan and lipophosphatidic acid, subsequently triggering prophenoloxidase activation and antimicrobial peptide synthesis [90]. This study elucidated the molecular mechanism of antimicrobial peptide production, while a study by Geijtenbeek et al. revealed how pathogens inhibit the host’s antimicrobial peptide response by targeting specific receptors. This finding provides new insights into the immune escape mechanism of pathogens [91].

These findings deepen our understanding of the antimicrobial peptide system in insects, demonstrating its multiple functions in immune defense. Future studies are needed to further explore the mechanism of action of antimicrobial peptides, especially regarding how to optimize their defensive efficacy in the face of novel pathogens. This is important for the development of novel biopesticides and disease control strategies.

### 4.4. Reactive Oxygen Species (ROS) and Double Oxidase (Duox) Systems

Insects rely on ROS/Duox for immunity, where TLR5 detects flagellin and induces ROS as a frontline defense [92].The Oliveira study further clarified the relationship between nucleic acid perception and ROS production, revealing how pathogenic nucleic acids trigger the oxidative stress response [93]. Jentho and Weis’s studies on damage-related molecular patterns (DAMPs) and innate immune training have shown that ROS production not only directly participates in the elimination of pathogens, but also regulates the formation of immune memory [94]. The role of ROS in aseptic inflammation has been investigated, and in one study it was found that reactive oxygen species have important functions in sensing and responding to injury [95]. This study specifically underscores the Duox pathway’s vital function in regulating gut homeostasis. Through systematic analysis, Gong et al. demonstrated that in sterile inflammatory conditions, DAMP receptor stimulation is intimately associated with increased ROS production [96]. Matzinger revealed the role of ROS signaling in the recognition of potential threats by the immune system by studying immune tolerance and danger signaling [97]. This finding provides new insights into how insects maintain tissue homeostasis using the ROS-Duox system. Recent studies by Hrithik et al. have shown that damage signaling triggers an immune response caused by the release of DAMP molecules during *Bacillus thuringiensis* infection, and that ROS production is a key link in this process [98].

**Figure 2:**
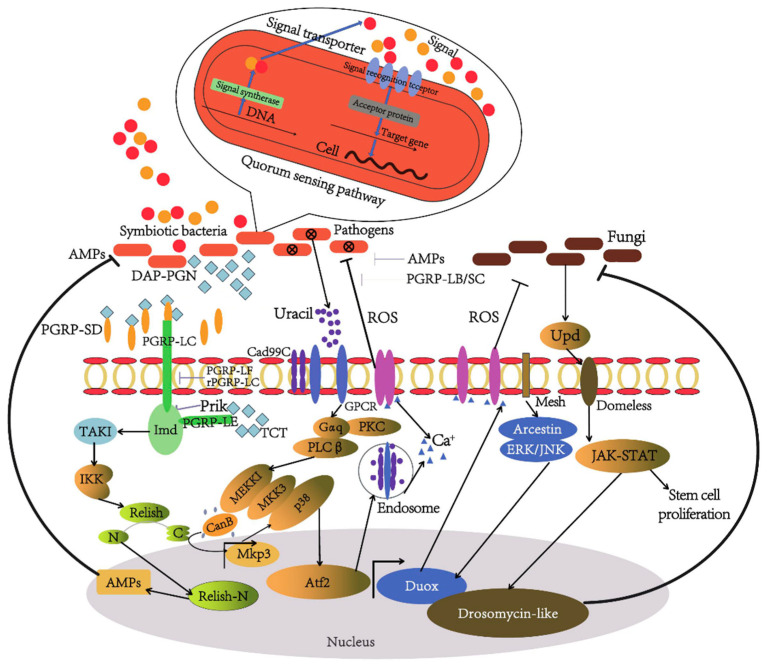
Illustrates multiple immune signalling pathways within the insect gut, including the IMD pathway, Toll pathway, and JAK-STAT pathway. These pathways are activated through various pattern recognition ‘receptors (such as PGRP-LC), ultimately inducing the production of antimicrobial peptides (AMPs) to defend against pathogen invasion. Furthermore, the figure depicts the generation of reactive oxygen species (ROS) and their pivotal role in host defence. The upper portion of the diagram elucidates the mechanisms by which symbionts, pathogens, and their interactions influence the host immune response, including microbial community interactions regulated by quorum-sensing signals.

The progress made in this area has deepened our knowledge of how the ROS-Duox system functions in the insect immune response and has also provided the basis for developing pest control methods that focus on regulating oxidative stress. It is important for future studies to delve deeper into the specific regulatory mechanisms of the ROS signaling network and explore how this knowledge can be used to enhance insects’ resistance to disease. This research direction will aid in the creation of more effective and environmentally friendly pest control methods. Figure 2 shows the dynamic regulation and functional evolution of the insect gut microbiota during host development [99].

## 5. Potential Applications of Insect Gut Microbiota: Agriculture

### 5.1. Agricultural Applications

The insect gut microbiota represents a promising frontier in agricultural biotechnology, offering innovative solutions for sustainable agriculture and crop protection. Recent research has demonstrated the significant potential of insect gut microorganisms for use in various agricultural applications, particularly in pest management, crop protection, and sustainable feed production [100]. The symbiotic relationships between insects and their gut microbiota have prompted the evolution of complex mechanisms that can be harnessed for agricultural enhancement. This mechanism provides scientific justification for the application of insect resources in sustainable animal feed production, helping to reduce traditional agriculture’s reliance on chemical additives and promoting the establishment of ecological agricultural systems [101]. In sustainable pest management, the insect gut microbiota has emerged as a powerful tool for developing eco-friendly control strategies. The microbiome-mediated interactions between insects and plants provide novel insights into pest control mechanisms, offering alternatives to conventional chemical pesticides. Recent studies have revealed that manipulating the gut microbiota of pest insects can significantly affect their fitness and host plant adaptation capabilities, presenting opportunities for innovative pest management approaches [102]. This review article systematically outlines how insects—particularly black soldier flies and mealworms—can be utilised to process agricultural and food waste, converting it into high-value products and ultimately achieving sustainable agriculture. The paper emphasises the pivotal role played by insect gut microbiota in this transformation process [103]. This review emphasises that insect bioconversion technology, as a green technology for transforming agricultural waste into valuable resources, holds considerable promise. This technology has successfully established an economic model consistent with the principles of circular agriculture [104].

Moreover, the role of insect gut microbiota in sustainable crop protection extends beyond direct pest control. The vast symbiotic bacterial communities residing within insects represent a largely untapped reservoir. These microorganisms produce an extraordinarily diverse array of secondary metabolites (natural products) possessing unique biological activities. Such compounds hold immense potential for application across agriculture, medicine, and other fields [105].

### 5.2. Environmental Governance

The application of insect gut microbiota in environmental management represents a significant advancement in sustainable biotechnology solutions. Recent studies have revealed that these microbial communities possess unique enzymatic capabilities that can effectively degrade complex organic pollutants and transform environmental contaminants [106]. The insect gut represents an exceptionally rich and under-explored repository of resources, harbouring microorganisms and enzymes with immense biotechnological potential [107].

The symbiotic microorganisms of insects—including gut microbiota and intracellular symbionts—constitute a vast and largely untapped resource pool, possessing revolutionary potential for the development of next-generation biotechnology products [108]. This study represents an in-depth exploration and validation of this resource repository: a unique alkalophilic bacterium was isolated from the gut of the domesticated silkworm, confirming that this strain can serve as an efficient microbial cell factory for converting biomass into lactic acid. This highlights the immense potential of insect symbionts for application in industrial biotechnology [109].

Moreover, the gut microbiota of insects plays a pivotal role in developing novel biotechnological processes for waste stabilisation. This study comprehensively elucidates the intricate and diverse symbiotic relationships between insects and their internal microorganisms—encompassing both gut microbiota and intracellular symbionts—with particular emphasis on the ecological drivers of these interactions, their maintenance mechanisms, and their profound biological significance for the survival and evolution of insect hosts [110]. The integration of these microbial systems in environmental biotechnology represents a promising approach for addressing contemporary environmental challenges while promoting sustainable industrial practices.

### 5.3. Industrial Biotechnology

The industrial applications of insect gut microbiota have emerged as a significant frontier in biotechnology, offering innovative solutions for sustainable manufacturing processes. These microorganisms harbor diverse enzymatic systems capable of catalyzing various industrial transformations, particularly in the production of novel biomaterials and specialty chemicals. Recent research has demonstrated their exceptional potential in producing industrial enzymes, including cellulases, lipases, and proteases, which have applications across multiple sectors.

The biotechnological development of insect gut microbiota has revealed its immense application potential in industrial enzymatic processes. Research indicates that these microorganisms possess unique metabolic capabilities to convert complex substrates into high-value products, thereby opening entirely new pathways for industrial bioconversion [111]. The study further indicates that these symbiotic systems and metabolic pathways, optimised through natural evolution, hold immense application potential in the fields of synthetic biology and biotechnology [112].

Furthermore, advances in our understanding of the molecular mechanisms of insect gut microbiota have led to the development of innovative bioreactor systems. The integration of these microbial resources into industrial biotechnology not only enhances the production efficiency but also contributes to the development of sustainable manufacturing practices, marking a significant advancement in bio-based industrial processes.

### 5.4. Human Health

The exploration of insect gut microbiota offers promising potential for their use in human health applications, particularly in nutrition and therapeutic development. Recent studies have demonstrated that insect-derived products, enriched with beneficial gut microorganisms, can serve as valuable sources of dietary fiber and bioactive compounds that contribute to human nutrition [113]. Yellow mealworms (Tenebrio molitor) are regarded as a highly promising sustainable animal feed source to replace fishmeal and soybean meal, owing to their exceptional nutritional value (high protein content and richness in unsaturated fatty acids) and their capacity to promote a healthy gut microbiome, It has been demonstrated to support multiple aspects of human health [114].

The gut microbiome of edible insects has garnered significant attention for its potential to enhance the nutritional value and safety of insect-based foods. As this review emphasises, the value of edible insects extends far beyond providing basic proteins and nutrients (i.e., “beyond human nutrition”); they also harbour immense potential for promoting health (health benefits) [115]. Furthermore, investigations into the chitin content of insect exoskeletons and their associated gut microbiota have unveiled potential applications in developing functional food ingredients and dietary supplements. These components show promising prebiotic properties and can positively influence the composition and function of the human gut microbiota. The incorporation of insect-derived products and their associated microorganisms into human nutrition is an innovative approach to achieving nutritional security and promoting the achievement of health goals [116].

**Figure 3:**
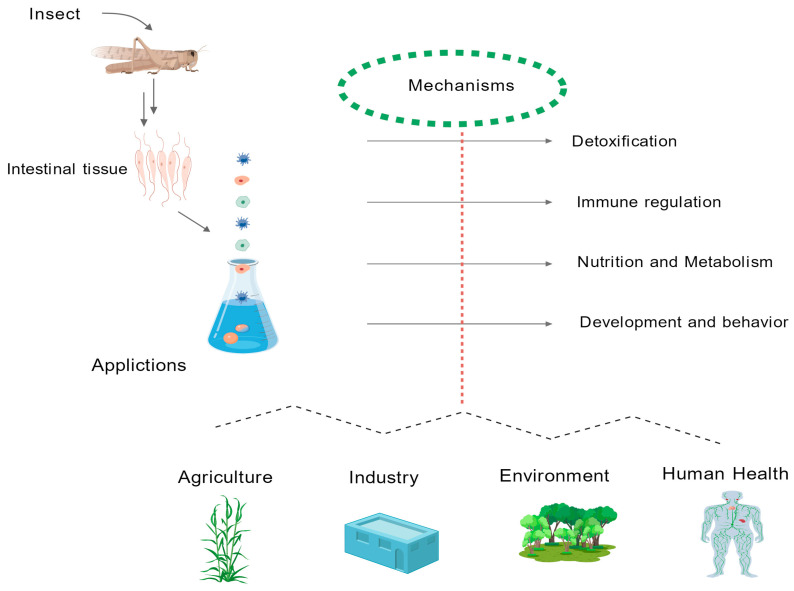
Innate immune defense mechanisms in the insect gut regulate the composition and function of the colonizing microbiota. This graphic was generated using the General-Purpose High-Quality Biomedical Graphics Database (GDP), a comprehensive repository of standardized biomedical visualization tools [17].

Figure 3: Interactions between an insect’s intestinal immune system and its microbial community (graphical summary).

## 6. Conclusions

The gut microbiota of insects demonstrates potential applications across multiple fields: by regulating the microbiota, biological pest control in agriculture and health management of pollinating insects can be achieved; its unique detoxification and degradation capabilities offer new insights into the bioremediation of organic pollutants, heavy metals, and plastic waste; as a valuable source of industrial enzymes and antimicrobial agents, it can drive the development of biomanufacturing technologies; and simultaneously, research into the mechanisms of its interaction with the host provides important references for human gut microbiome studies. Research into insect gut microbiota has revealed a complex and sophisticated microecosystem, demonstrating profound interactions between microorganisms and their hosts Research on insect gut microbiota still faces numerous challenges. For instance, efficiently decodironment interactions remain pressing issues. Additionally, technological limitations pose difficulties in functional validation and application development, necessitating the introduction of more systems biology and synthetic biology approaches for precise control and optimization of microbial function.

Future research directions should focus on (1) employing high-throughput omics technologies and systems biology approaches to comprehensively analyze microbiome functions and explore specific regulatory mechanisms in hosts’ physiological processes; (2) utilizing synthetic biology techniques to develop and optimize microbial communities for addressing specific agricultural and environmental challenges; (3) strengthening multidisciplinary collaboration, integrating the latest advances in ecology, microbiology, and biotechnology to jointly promote innovation and breakthroughs in insect gut microbiota research. Other areas for future research include the application of metagenomics/Metatranscriptomics to decode microbial metabolic networks and, when combined with host transcriptomics, to reveal cross-kingdom signaling pathways; synthetic biology applications, utilizing CRISPR-based tools to engineer microbial communities for targeted pollutant degradation, enhanced nutrient acquisition in agricultural systems, development of standardized host strains (e.g., *Bacillus* spp.) for functional validation, and translational medicine based on the comparative responses of insect and human microbiomes to exogenous substances; adapting insect-derived antimicrobial peptides for clinical applications; and establishing a shared database for cross-species microbiome analysis.

In conclusion, research on insect gut microbiota not only deepens our understanding of insect biology, but also provides novel approaches to addressing global challenges in agriculture, the environment, and health. As this field continues to evolve, innovative biotechnology applications based on insect gut microbiota will undoubtedly play an increasingly significant role in the future.

## Data Availability

The content of this article is entirely based on a comprehensive synthesis of published literature, with all cited studies duly referenced.
